# Unmasking Determinants of Specificity in the Human Kinome

**DOI:** 10.1016/j.cell.2015.08.057

**Published:** 2015-09-24

**Authors:** Pau Creixell, Antonio Palmeri, Chad J. Miller, Hua Jane Lou, Cristina C. Santini, Morten Nielsen, Benjamin E. Turk, Rune Linding

**Affiliations:** 1 Department of Systems Biology, Technical University of Denmark, 2800 Lyngby, Denmark; 2 Centre for Molecular Bioinformatics, University of Rome Tor Vergata, 00133 Rome, Italy; 3 Department of Pharmacology, Yale University School of Medicine, New Haven, CT 06520, USA; 4 Biotech Research & Innovation Centre (BRIC), University of Copenhagen (UCPH), 2200 Copenhagen, Denmark

## Abstract

Protein kinases control cellular responses to environmental cues by swift and accurate signal processing. Breakdowns in this high-fidelity capability are a driving force in cancer and other diseases. Thus, our limited understanding of which amino acids in the kinase domain encode substrate specificity, the so-called determinants of specificity (DoS), constitutes a major obstacle in cancer signaling. Here, we systematically discover several DoS and experimentally validate three of them, named the αC1, αC3, and APE-7 residues. We demonstrate that DoS form sparse networks of non-conserved residues spanning distant regions. Our results reveal a likely role for inter-residue allostery in specificity and an evolutionary decoupling of kinase activity and specificity, which appear loaded on independent groups of residues. Finally, we uncover similar properties driving SH2 domain specificity and demonstrate how the identification of DoS can be utilized to elucidate a greater understanding of the role of signaling networks in cancer (Creixell et al., 2015 [this issue of *Cell*]).

## Highlights

• Residues driving specificity in the kinase and SH2 domains are globally identified
• Three new such residues, termed αC1, αC3, and APE-7, are experimentally validated
• Specificity and catalytic activity appear to be encoded in distinct sets of residues
• The global identification of determinants allows the modeling of rewiring mutations

## Graphical Abstract

**Figure:**
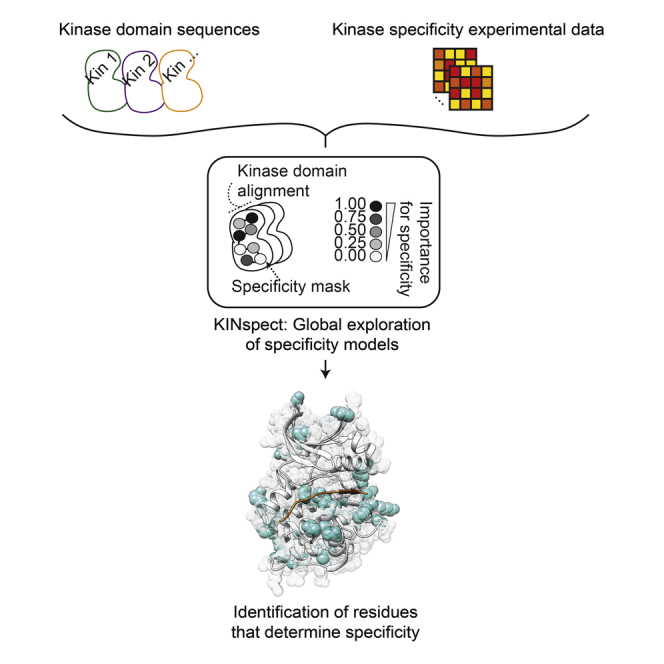

## Teaser

Determining the residues that drive the specificity of kinases and of SH2 domains that bind phosphorylation sites paves the way for a systematic interpretation of mutations on signaling networks.

## Introduction

Cellular organization and response to external and internal cues relies on swift and precise processing of information through cell signaling networks. High fidelity in these circuits depends critically on the recognition and phosphorylation of specific substrates by protein kinases, and perturbations of this cellular system have been linked to significant evolutionary transitions (Capra et al., 2012, Skerker et al., 2008, Tan et al., 2009, Zarrinpar et al., 2003), as well as to disease progression, in particular, in cancer (Borrello et al., 1995, Creixell et al., 2012, Marengere et al., 1994, Santoro et al., 1995, Songyang et al., 1995).

**Figure 1:**
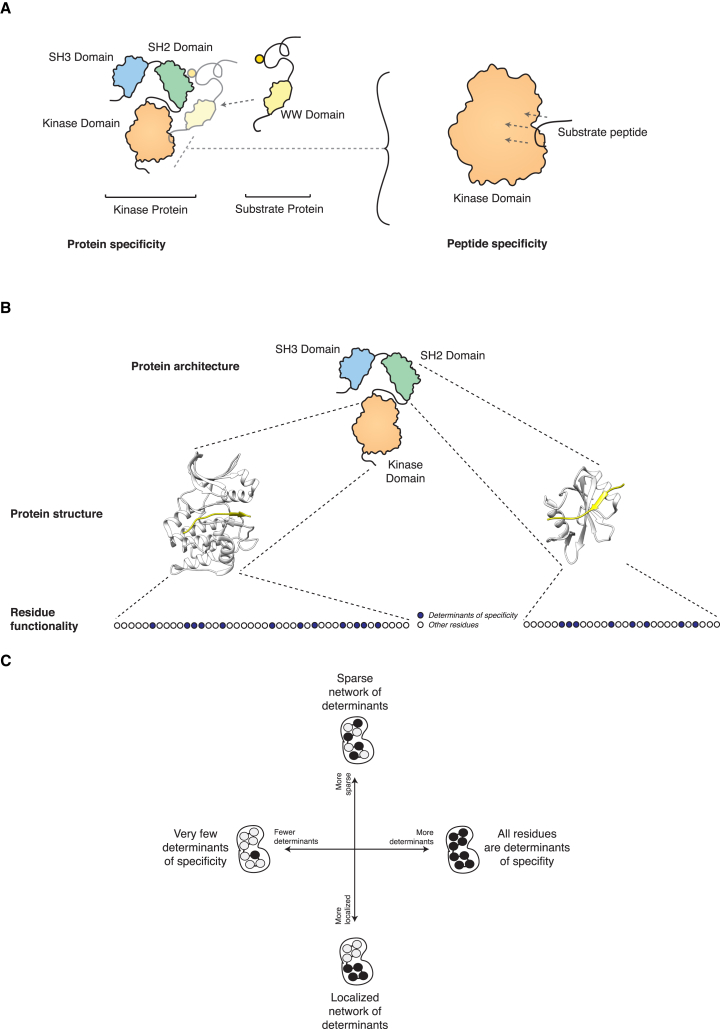
Open Questions in Protein Domain-Peptide Specificity (A) Protein specificity determines the interaction between the whole kinase protein and its substrates and is driven by processes such as interactions between other domains and motifs (e.g., SH2 and phospho-tyrosine in this figure), co-expression of the two proteins, cellular localization, scaffold proteins, etc. (Bhattacharyya et al., 2006, Linding et al., 2007, Reményi et al., 2005, Scott and Pawson, 2009). Peptide specificity, in contrast, is solely driven by the sequence and structure of the kinase domain and drives the phosphorylation of specific linear motifs within the substrate protein. (B) The so-called determinants of specificity (DoS) are those residues within a protein domain that together drive and determine the peptide specificity of the domain. (C) While relatively few localized DoS have been described in the kinase domain, this study explores the existence of more determinants and their relative domain positions.

**Figure S1:**
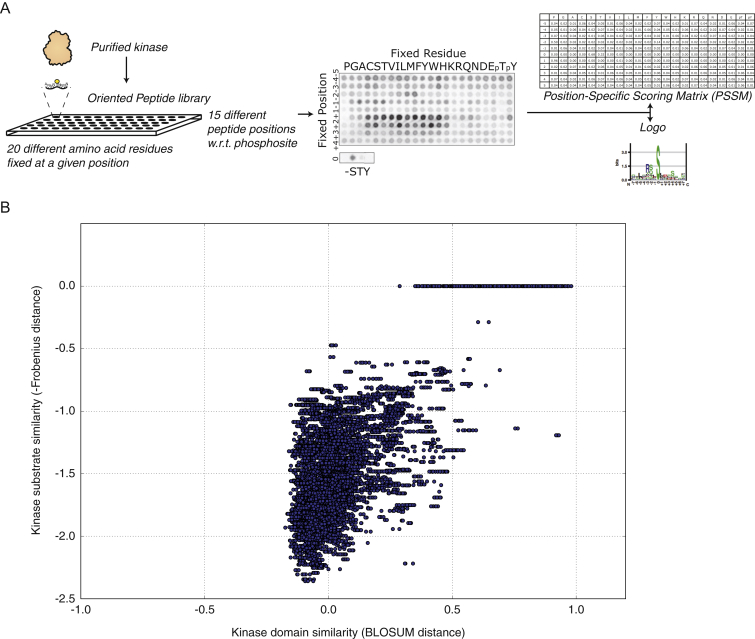
Related to Figure 1 (A) Experimental Determination of the Peptide Specificity of a Kinase Domain. Peptide specificity is determined experimentally by Positional Scanning Peptide Library (PSPL) screening (Hutti et al., 2004), where purified kinases are exposed to random peptides where only specific positions are fixed to one particular amino acid residue, thus determining substrate molecular preferences for every given kinase. The experimental results can be turned into Position-Specific Scoring Matrices (PSSM) or motif logos. NetPhorest (Miller et al., 2008) is a comprehensive collection of PSSMs (obtained from PSPL as well as phosphorylation motifs obtained by training artificial neural networks) for different domains in different species and the full human kinome collection covering 166 kinases was used in our study. Note that the data, PSSM, and logo were shown and included for illustration purposes only, and thus its values should not be treated as actual data. (B) Kinase Domain Similarity Does Not Correlate With Substrate Similarity. After collecting all human kinases for which substrate information (i.e., Position-Specific Scoring Matrices or PSSMs) is available, the domain similarity and substrate specificity similarity have been computed as the BLOSUM distance (from the BLOSUM62 matrix [Henikoff and Henikoff, 1992]) and, in order to measure similarity instead of dissimilarity, the negative of the Frobenius distance, respectively. As shown in the figure, strong direct correlation between sequence similarity at the whole domain level and substrate specificity similarity does not exist, indicating that substrate specificity is unlikely to be encoded by the entire domain. This observation prompted the introduction of specificity masks (different combinations of residues with different degrees of contributions to domain specificity) used subsequently in this study.

Cellular signaling fidelity is maintained essentially through two coupled mechanisms. At a macro-molecular level, protein specificity ensures that each protein kinase will reach and interact with its protein substrates. At a micro-molecular or atomic level, peptide specificity defines the ability of a given kinase domain present in all active protein kinases to recognize and phosphorylate a specific peptide within the protein substrate (Turk, 2008) (Figure 1A). A variety of experimental techniques have been developed to elucidate the peptide specificity for many modular signaling domains and obtain specificity profiles (e.g., the so-called Position-Specific Scoring Matrices, PSSMs), as a quantitative measure of the preference of each kinase domain for each amino acid residue at every peptide substrate position (Figure S1). While other factors contributing to protein interaction specificity at a macro-molecular level (such as co-localization, co-expression, docking motifs, and scaffold or adaptor proteins) have been described (Bhattacharyya et al., 2006, Linding et al., 2007, Reményi et al., 2005, Scott and Pawson, 2009), the combination of residues in the kinase domain that encode peptide substrate specificity, the so-called determinants of specificity (DoS), have remained largely elusive (Figure 1B). Even though some structural studies have helped identify residues that are in close contact with the substrate peptide which likely influence specificity (Brinkworth et al., 2003, Ellis and Kobe, 2011, Hanks and Hunter, 1995, Mok et al., 2010, Nolen et al., 2004), these studies were largely focused on specific kinase families and/or non-human species as well as limited in scope by the small number of kinase-peptide structures currently available and an inability to capture potentially long-range DoS.

Here, we present a computational approach that aims to overcome these limitations and address the following open questions. Which residues within the kinase domain contribute to peptide specificity (constituting the so-called DoS)? Are these determinants just a small group of residues localized in close proximity to the substrate as currently thought, or do they form a sparse network of residues instead (Figure 1C)? Are such principles of domain-peptide specificity conserved in other domains? Finally, how do these DoS relate, spatially and functionally, to those residues known to be involved in the regulation and catalytic activity of the kinase domain? In other words, are these different functionalities loaded onto the same residues or on independent groups of residues, and how did they evolve?

As we demonstrate in our accompanying article (Creixell et al., 2015 [this issue of *Cell*]), which explores how cancer mutations affect domain specificity by integrating the DoS identified here, resolving these questions could represent a valuable contribution not only for basic signaling biology but also for cancer research.

## Results

### Learning about Residue Contributions to Specificity by Sampling over Different Specificity Masks

**Figure 2:**
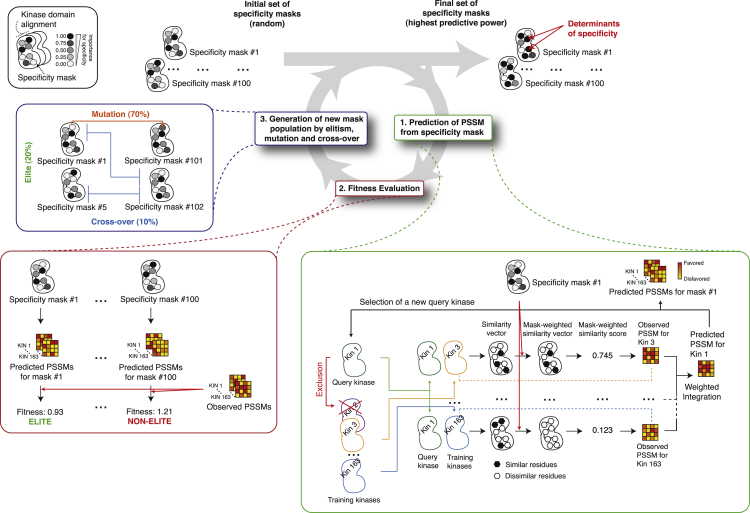
Overview of the KINspect Algorithm The KINspect workflow is designed to identify the specificity mask that best describes the importance of the different residues for specificity. Different combinations of contributions to specificity by different kinase domain residues are collected as specificity masks (top left), where a score between 0 and 1 is given to each position within the kinase domain. Originally, the specificity masks are initialized with random values to then follow a machine-learning procedure that will ensure the masks with the highest predictive power toward specificity are selected for and optimized. This procedure, known as a learning classifier system, is divided into three separate steps. In step 1, for each specificity mask the system loops over all query kinases and, using a kinase domain alignment, compares the query kinase to all other kinases (except those belonging to the same kinase family, which are excluded only at this stage to avoid over-fitting) at the sequence level, generating a similarity vector. This vector is combined with the specificity mask, so that similarity in high-scoring positions of the mask is reinforced and similarity in low-scoring position of the mask is silenced, effectively producing a mask-weighted similarity vector and sum score for each kinase. These values are subsequently used to integrate the different observed PSSMs into a combined predicted PSSM for the query kinase (as further explained by the equations and text in Supplemental Experimental Procedures section and in Zhang et al., 2009). In step 2, after a predicted kinase has been generated for all the kinases in our set, fitness is computed as the median of all the differences between the predicted and the experimentally determined PSSM for all the kinases obtained from the NetPhorest repository (Miller et al., 2008). In step 3, the best-performing specificity masks are kept (“elite”), and new ones are generated by mutation (changing the value of a given position in the mask) and cross-over of the elite sequences (combining two segments of two other masks), as typically done in genetic algorithms. Once a new set of masks has been generated, the whole procedure (prediction, fitness evaluation, and generation of new masks) is repeated iteratively until fitness (defined as median error between predicted and observed specificity profiles) cannot be improved any further (i.e., convergence is reached). Residues scoring high in the optimized specificity masks will be considered candidate DoS. For further details on this procedure, please refer to Supplemental Experimental Procedures.

When investigating the relationship between kinases at the domain primary sequence similarity level and at the substrate sequence motif similarity level (using specificity profiles or PSSMs derived from Positional Scanning Peptide Library or PSPL experiments, see Experimental Procedures and Figure S1), it is apparent that, when considering the domain in its entirety, no strong linear correlation between these exists (Figure S1). We hypothesized that this lack of correlation could indicate that substrate specificity is not encoded by the domain as a whole. Instead, we hypothesized that a limited number of residues contribute to specificity, and that those that do contribute, are likely to do so to different degrees. In order to capture this principle, we introduced the specificity mask as a fundamental entity in our approach. As depicted in Figures 1B and 2 (small box), a specificity mask is defined as a particular combination of contributions to specificity from the different residues in the kinase domain. For example, an extreme hypothesis where all residues within the kinase domain contribute equally to specificity would be represented by all entries in a mask with the same score (e.g., 0.5). Instead, a situation where a single residue, X, would drive specificity would be represented by all entries scoring 0.0 except position X scoring 1.0.

Our approach (described below) explores the possibility that within a large ensemble of specificity masks, certain masks can discriminate between kinases with dissimilar substrate specificities better than others. These masks will range from those capturing very few and localized DoS (reminiscent of models explored in the structural studies; Brinkworth et al., 2003, Ellis and Kobe, 2011, Hanks and Hunter, 1995, Mok et al., 2010, Nolen et al., 2004) to those capturing a larger number of determinants distributed more sparsely across the kinase domain (Figure 1C). As further detailed in the next section, since our aim was to identify new DoS following an unbiased data-driven systematic approach, we did not impose any restrictions in the set of specificity masks that can be found; instead, we explore a large set of possible specificity masks and let the system evolve and find those showing the best discriminatory capabilities.

### The KINspect Methodology

In order to identify which residues contribute to specificity, we developed a computational framework named KINspect, which explores a very large number of combinations of residues, and their contribution toward specificity, and subsequently identifies those featuring the best predictive capability (Figure 2). This type of approach, known in machine learning as learning classifier systems (Lanzi et al., 2000), enables the selection of the best-performing set of specificity masks starting from a large initial set of random masks by following three consecutive steps (Figure 2).

First, for each specificity mask, the specificity profiles (PSSMs) for each kinase are predicted by comparing all kinases across the human kinome at each amino acid position within the kinase domain (amino acid similarity) and by incorporating a weighting factor (from 0 to 1; 0 being not important, 1 being critical) of the “specificity importance” of each position as determined by the given specificity mask. A PSSM for each kinase is then predicted by integrating the PSSMs for the other kinases using the mask-dependent similarity as a weighting factor. Naturally, the majority of masks within the original set of random masks will predict specificity poorly, but, as the system evolves, the masks will improve their predictive power, i.e., become more fit.

Second, masks are ranked according to their predictive performance (i.e., their ability to predict PSSMs that are similar to the experimentally determined PSSMs). In essence, masks that more closely capture the true contribution of each position within the kinase domain (i.e., those scoring higher at kinase domain positions that truly contribute to specificity) will result in a better prediction of the specificity profiles, thus ranking higher.

Third, the worst-performing masks are filtered out and new masks, representing both subtle (mutation) but also more abrupt (cross-over) variations of the best-performing masks, will be added.

These three steps are initially started with random specificity masks and repeated until convergence is reached and fitness cannot be optimized further. Residues consistently scoring higher in the specificity masks following the optimization procedure will be considered candidate DoS. For a more technical description of the algorithm, please refer to Figure 2 and Extended Experimental Procedures.

### Model Robustness, Validation, and Coverage

**Figure S2:**
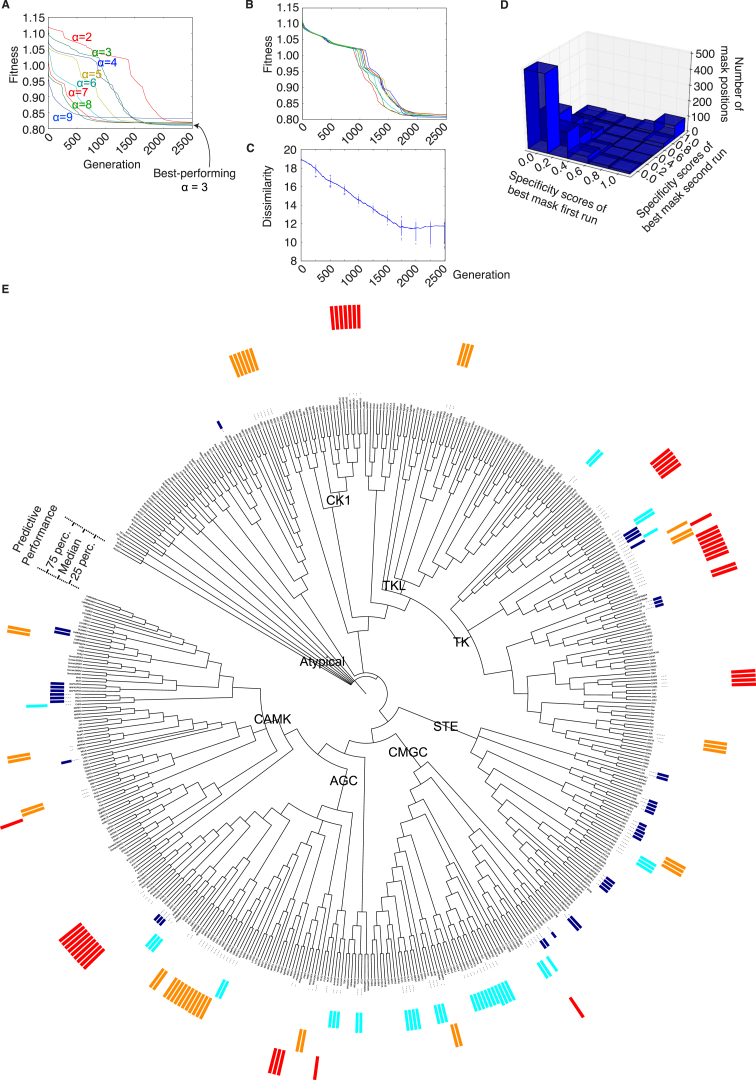
Related to Figure 2 (A) Alpha Determination for Kinase KINspect. As explained in Experimental Procedures, a parameter ‘alpha’ (α) needs to be optimized to determine the best trade-off between using only the most similar domains or include more distant domains when predicting new PSSMs. In essence, the procedure described for KINspect in Figure 2 is performed using different alphas and the alpha leading to the best performance is chosen. As shown here, the best results (lower prediction error) were obtained with α = 3, thus this value was used subsequently. Even though, in line with standard nomenclature for genetic algorithm, we have labeled the y axis as being “Fitness,” it is important to clarify that KINspect evolves by minimization the error in predictions, therefore “minimizing fitness.” This “Fitness” is measured as the median Frobenius distance between predicted and experimentally determined PSSMs. (B) KINspect fitness trajectories. When trained on the human kinome, KINspect reaches convergence after approximately 2000–2500 generations. Fitness is measured as the median Frobenius distance between predicted and observed PSSMs. Each color in this plot shows the fitness of the best mask at each generation. The similarity between the different trajectories representing the 10 independent KINspect evaluation runs confirms they have followed a similar path to convergence. (C) KINspect convergence, robustness and performance. In order to evaluate whether similar results are obtained in the 10 independent KINspect evaluations, the best mask for each run is compared to all the others at each generation and their dissimilarity is measured as the Frobenius distance between the vectors. By including box-plots every 500 generations, we could also assess the evolution of the overall distribution. The graph illustrates the increase in similarity (decrease in dissimilarity) of results as one moves closer to the final point of convergence. From this, one can conclude that independent algorithm deployments tend to converge to the same (or at least highly similar) solution. One can further appreciate the similarity corresponding to this Frobenius distance by referring to (C), where the scores of two masks at this distance are represented pair-wise. (D) By comparing two of the final specificity masks obtained in two independent KINspect evaluations, we could compare the score of the two masks at the same kinase domain positions. This distribution shows a large degree of agreement (e.g., residues scoring 1 in one masks have a high tendency to score 1 in the other one) between the two final masks obtained in two independent KINspect evaluation runs, as well as a strong tendency for most residues to score 0 in both runs. (E) KINspect coverage. Overview of the predictive performance of KINspect for different human kinase domains. A larger bar indicates higher (better) predictive performance, while a shorter bar indicates lower (worse) predictive performance. For more clarity, bars have been colored in dark, light blue, orange or red (predictive performance below the percentile 25, below the median, above the median or above the percentile 75, respectively).

**Figure S3:**
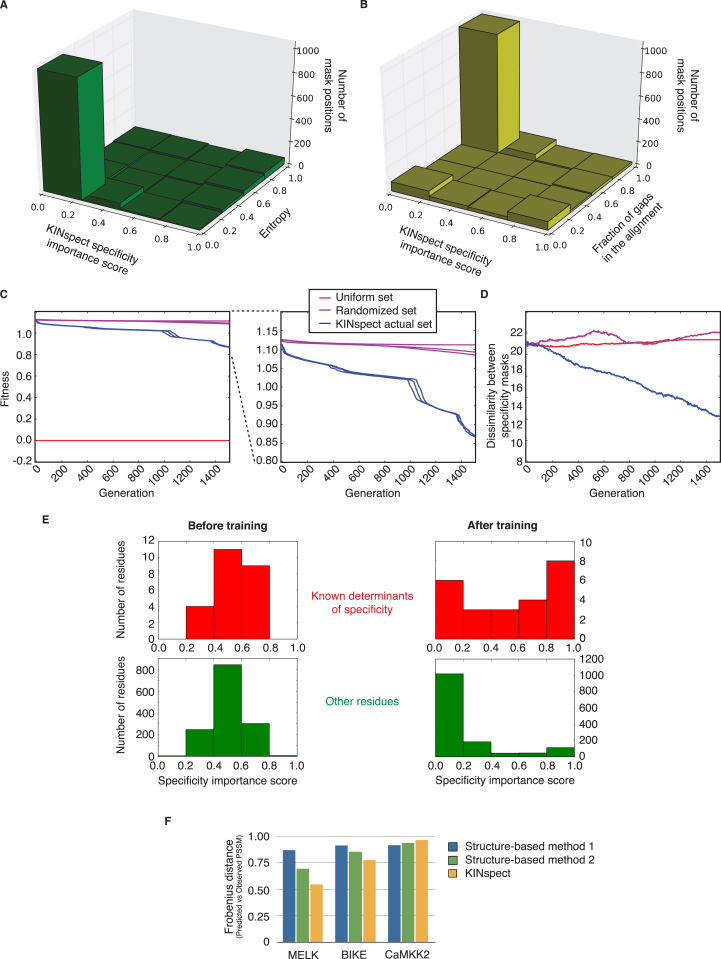
Related to Figure 3 (A and B) The DoS Identified by KINspect Cannot Be Explained Simply By Conservation (A) or Alignment Gaps (B). In order to refute the possibility that we are simply identifying the most conserved positions in our alignment or that gaps in our alignment bias substantially our results, we plotted alignment column entropy (A) and the gap fraction (B) versus the position score. These results confirm that neither conservation (i.e., lower entropy) nor alignment gaps would directly explain our findings, thereby demonstrating the robustness of our method to such potential artifacts. (C and D) Randomized or Uniform Versions of Our Sequence-Specificity Sets Do Not Result In Optimized Convergent Results. In order to confirm that our results are not a result of intrinsic properties of our method or somehow uncoupled from our data, we produced two control set; one with all specificity profiles set to the same uniform matrix (Uniform set) and a second one, where the linkage kinase-specificity profile was randomized (Randomized set). Neither of these two control sets leads to an optimization process (i.e., decrease in fitness landscape terms) similar to the one observed for the actual KINspect set, represented in blue. Note that the uniform set does not effectively represent a predictive challenge for the method, which explains why the fitness remains at 0.0 for all iterations. In addition to this marked decrease in optimization potential, unlike in the actual set, the two control sets do not lead to convergent masks either (i.e., the dissimilarity between the masks is kept high along the optimization process), as observed in (D). (E) Enrichment in Previously Reported DoS. In order to investigate whether the specificity mask identified previously described determinants of specificity, we curated from the literature a number of determinants identified using different means and in different species (Table S1). Next, we compared the KINspect score obtained by this group of previously described determinants (top) as well as all other residues (bottom) at the beginning of the evaluation run (left, before optimization) and after KINspect was optimized (right). Marked different distribution trajectories can be observed between both groups, with most residues tending toward zero at the bottom, while a much larger fraction of residues previously identified as determinants score higher at the top, illustrating an enrichment of previously reported DoS (Fisher’s exact test one-sided, p = 8.4x10^−7^). Interestingly, several additional DoS were identified by KINspect (bottom right) and some of the reported DoS did not obtain a high KINspect score (some of which had been reported in non-human species). (F) Comparison to Previous Methods. Whereas a global comparison to previous methods would be unfeasible due to the highly limited coverage of human kinases that previous methods utilized, we were able to employ the gold standard set used in the DREAM challenge on peptide specificity (Ellis and Kobe, 2011). While KINspect performed similarly poorly on CaMKK2 (a kinase with very distinct specificity), we could confirm in this limited test set that KINspect outperforms previous structure-based methods (Brinkworth et al., 2003, Ellis and Kobe, 2011) in its ability to predict PSSMs that are close to the experimentally observed ones (p = 2.20x10^−47^, 4.58x10^−27^ and 3.08x10^−04^ for MELK, BIKE and CaMKK2, respectively).

Since our method contains stochastic aspects (such as the starting set of random masks and the generation of new masks by mutation and cross-over), one initial question that must be addressed is whether the method is robust to this initial stochasticity, i.e., whether one would obtain similar results if the process was started with arbitrary initial conditions and evaluated independently several times. To this end, we compared the fitness evolution of ten independent KINspect evaluations and found highly comparable fitness trajectories, as well as increasing similarity between the best-performing masks at each generation (Figure S2; Data S1, S2, and S3). Moreover, we confirmed that the results are not simply due to trivial technical factors, such as residue conservation or alignment gaps (Figure S3), and that similar results could not be obtained using uniform or randomized sets (Figure S3). Taken together, these results demonstrate that KINspect is robust to arbitrary initial conditions and converges to a limited set of highly similar solutions (specificity masks, Figure S3).

Moreover, we also explored a vast number of possible combinations of residues and specificity models. Since convergence in the model requires approximately 2,500 cycles of the above three steps (in the case of the human kinase domain) and 100 specificity masks are used at every generation, 250,000 models were explored in the kinome-wide search for the most informative masks. By repeating this algorithmic deployment independently ten times with arbitrary initial conditions, 2,500,000 models were explored in total. The high number of models explored and the fact that the independent evaluations converge on their solutions imparted confidence that the results obtained could be close to the “true mask” of specificity.

In order to further benchmark our approach, we collected an inclusive “golden list” of residues that had been suggested or predicted as DoS (Table S1) in the literature covering a variety of methods and species (Brinkworth et al., 2003, Hanks and Hunter, 1995, Johnson et al., 1998, Mok et al., 2010, Nolen et al., 2004) and explored the possibility that the best masks would be enriched in this set of “golden” determinants. Indeed, Figure S3 shows that, while the distributions over specificity scores of previously reported DoS and other residues are probabilistically equivalent at the start of the optimization process, they are remarkably different at the end of it, supporting the aforementioned enrichment (Fisher’s exact test one sided, p = 8.4 × 10^−7^).

In addition to identifying candidate DoS, our approach can predict the domain specificity (PSSM) of every kinase in the human kinome from sequence alone. Therefore, we could compare these to those kinases where the specificity profile has previously been experimentally determined (Miller et al., 2008) and assess the algorithm’s predictive accuracy (Figure S2). As shown in Figure S2, KINspect presents better sequence-specificity predictive capabilities for some families (e.g., CK1 group) than others (e.g., STE group), likely reflecting both biological differences and algorithmic preferences (for instance, particular family differences in specificity that could not be captured by our kinome-wide specificity masks). Finally, for a small set of kinases used as a “gold standard” in the DREAM challenge (Ellis and Kobe, 2011) and that, importantly, were not part of our training set, we could confirm that overall KINspect performed better than other methods (Figure S3).

**Figure 3:**
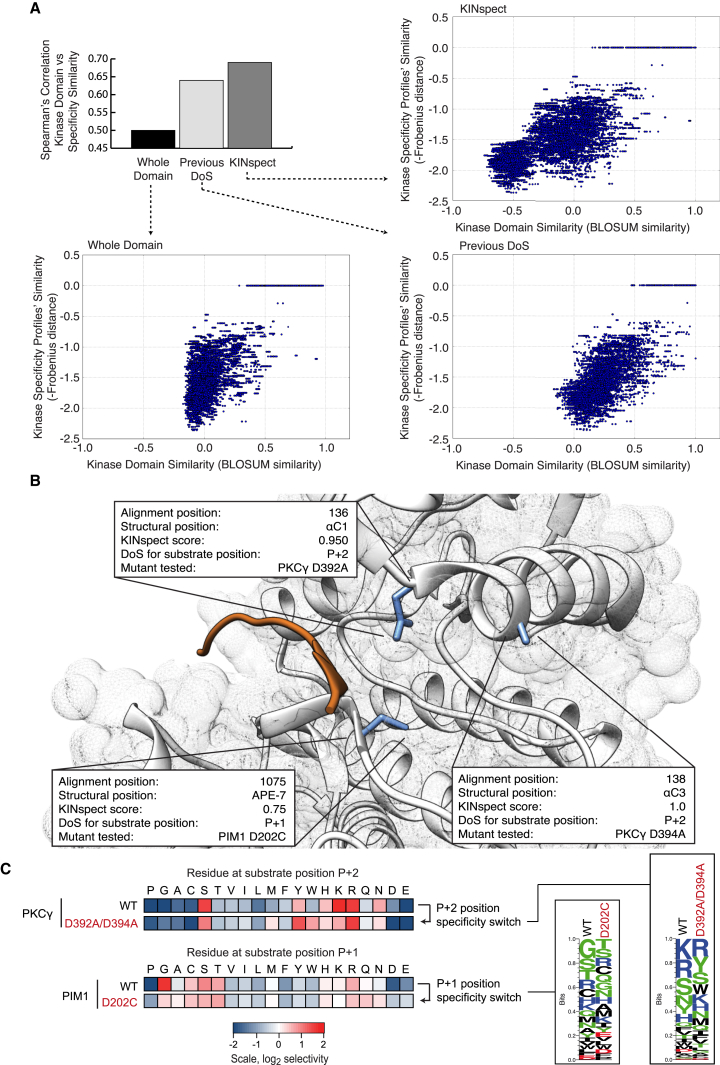
Computational and Experimental Validation of the DoS Identified by KINspect (A) Scatterplots comparing pairwise relationships between kinases’ domain sequences, and their specificity profiles can illustrate the lack or existence of correlation between sequence and specificity. By limiting the comparison to specific sets, one can investigate whether such sets encode for specificity (i.e., maintain or increase the correlation), as measured by Spearman’s correlation coefficients. By comparing the correlations obtained from different sets of residues, the whole domain on the left, previously reported determinants of specificity in the middle and KINspect scores on the right, we confirm that residues with a high KINspect score encode for specificity (e.g., residues scoring above 0.9 lead to very high sequence-to-specificity correlation, with a Spearman’s correlation coefficient of 0.69, despite representing only 5.73% of the residues in the kinase domain alignment). Further comparisons with other sets of residues can be found in Figure S4. (B) Three new candidate determinants of specificity predicted by KINspect, positioned in the first and third residues of the αC helix and seven residues before the APE motif delimiting the activation segment, are experimentally verified to encode specificity by PSPL as described in Experimental Procedures. (C) Experimental results for the PKCγ and PIM1 mutants showing a specificity switch for P+2 and P+1 substrate positions, as shown in matrix and logo form (logos generated using Seq2Logo; Thomsen and Nielsen, 2012). Complete PSSMs describing the PSPL results for wild-type and mutant kinases can be found in Figure S4.

**Figure S4:**
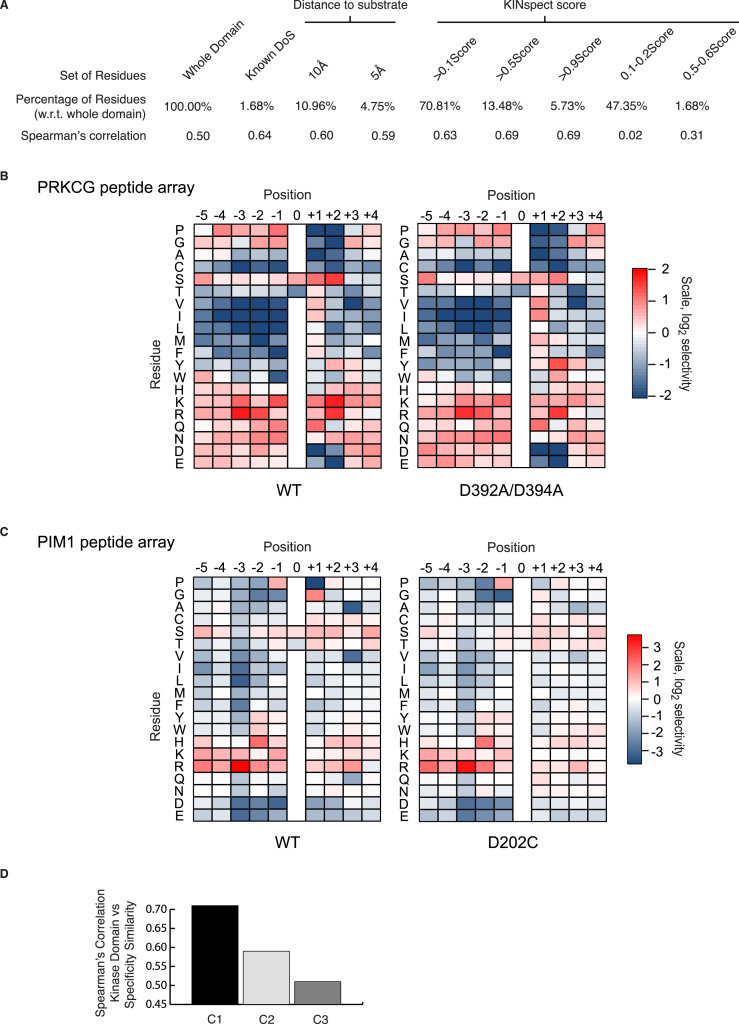
Related to Figures 3 and 4 (A) No Other Separation Method Leads to a Higher Sequence-to-Specificity Correlation than KINspect. As shown in the figure, the correlation varies between different subsets of residues, but is highest for residues that obtained high KINspect scores (KINspect Score > 0.9) despite being a relatively smaller fraction of domain alignment positions. (B and C) Complete PSSMs Obtained by PSPL for Wild-type and Mutant Kinase Experimentally Validating KINspect Predictions. Extended version of the PSSMs represented in Figure 3 showing the changes in peptide preferences when mutating three of the residues with high KINspect score. (D) Contribution to Specificity From Each Specificity Cluster. In order to quantify whether the different clusters of highly scoring residues contributed equally to specificity we computed the Spearman correlation using only residues belonging to each cluster. From the graph, it can be concluded that Cluster-1 encodes most of the specificity, in line with its closer proximity to and direct contact with the peptide substrate, followed by Cluster-2 and Cluster-3.

While the results in Figure S3E confirm enrichment in previously reported DoS, it is also important to note that KINspect identified a large number of additional DoS that had not been reported in the literature (e.g., 82 alignment positions above the arbitrary threshold of having a KINspect score above 0.8). Thus, we set out to evaluate the likelihood that these newly identified residues would be true DoS. Following up on our initial reasoning, we hypothesized that by identifying true DoS (the kinase domain residues that truly encode for the domain’s specificity) one should be able to observe better correlations between kinase sequence and kinase specificity, by limiting the comparison to this specific set of residues. Indeed, Figure 3A illustrates how limiting the comparison to those residues that obtained higher KINspect scores not only maintains, but, in fact, improves the sequence-to-specificity correlation by approximately 20% (as compared to the Spearman correlation obtained by considering the entire domain). Furthermore, we could confirm that other similarly small groups of residues, such as the set of previously reported DoS, or other selection strategies, such as residues close to the substrate, do not lead to similar improvements of the sequence-to-specificity correlation (Figure 3A; Figure S4).

We next selected a group of residues predicted by KINspect to be DoS and devised PSPL experiments to experimentally validate their involvement in specificity. In particular, as shown in Figure 3B, for our first experiment we selected two of the candidate DoS predicted by KINspect (named αC1 and αC3 as they are located on the first and third residues of the αC helix of the kinase domain) with scores of 1.0 and 0.95 that are in close proximity to residue P+2 in the peptide substrate. Next, since PKCγ has a strong preference for Arg and Lys at P+2 that had so far defied structural analysis, we mutated the αC1 and αC3 residues on PKCγ from the wild-type aspartates to alanines. As shown in Figure 3C (and Figure S4), the mutant form maintained the Arg preference but lost its Lys preference at this particular position, at the same time gaining preference for aromatic residues, thereby validating the specificity determining nature of these DoS predicted by KINspect.

For our second experiment, we selected a position (named APE-7 as it is located seven residues before the APE motif delimiting the activation segment) with a score of 0.75 in close proximity to residue P+1 (Figure 3B). Similar to the case of PKCγ in the αC1 and αC3 residues, Pim1 features an unexplained strong preference for Gly on position P+1, which is unusual for a kinase belonging to the CAMK family. Thus, we mutated Pim1 from its wild-type Asp to Cys, a residue more typically seen in other CAMK kinases, hypothesizing that if this single substitution could abrogate this Gly preference on position P+1, it would prove the specificity driving nature of the APE-7 residue. As shown in Figure 3C (and Figure S4), indeed this single-point mutation on Pim1 leads to a shift away from P+1 Gly preference to a non-specific profile similar to that of other CAMKs.

Taken together, these results demonstrate that KINspect successfully identified a set of residues on which the specificity of the entire domain is encoded.

### The Determinants Form Sparse Networks of Residues that Together Encode Specificity

**Figure 4:**
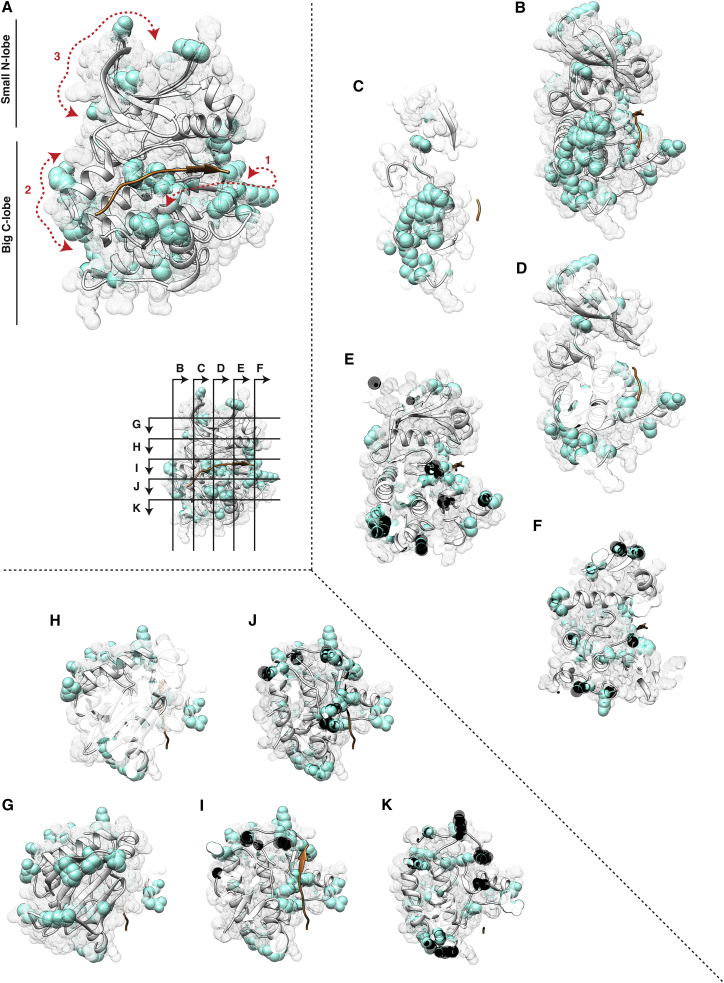
Determinants of Specificity in the Human Kinase Domain (A) Mesh representation of the kinase domain, including its secondary structure in cartoon representation and a bound peptide substrate colored in orange. Positions predicted as DoS by KINspect (i.e., residues with a KINspect specificity importance score higher than 0.9) are highlighted in cyan and the three “canals” formed by these determinants are outlined by red arrows. (B–K) For a more clear representation of different parts of the structure, longitudinal (B–F) and transversal (H–K) slices were taken through the kinase domain at the planes indicated in the inset of (A). A dynamic visualization of this structure can be found in Movie S1. The structure used is that of Akt/PKB in complex with GSK3 peptide (PDB ID: 1O6K; Yang et al., 2002), and the structural visualization on this and other subsequent figures was generated using Chimera (Pettersen et al., 2004).

In order to evaluate the relationship between the different DoS, as well as between the DoS and the peptide substrate, we investigated their spatial distribution in the kinase domain. Figure 4 and Movie S1 show the tertiary structure of the DoS identified by KINspect (alignment positions above the arbitrary threshold of having a KINspect score above 0.9 across ten independent deployments of KINspect) and offers two interesting observations:

First, we note that several of the determinants localize relatively far from the peptide substrate. However, most of these distant DoS seem coupled to other DoS through “canals” (i.e., existing structural paths connecting the different DoS among each other and ultimately with the substrate) that eventually contact the substrate peptide, as shown, for instance, in Figures 4B, 4C, or 4J. Such distribution of residues in networks spanning different domain sites and the presence of these “canals” suggest that specificity could possibly be encoded by groups of residues that communicate from different parts of the domain, perhaps in a similar manner to which other domains are regulated allosterically through protein sectors (Reynolds et al., 2011).

Second, closer inspection of the results (Figure 4; Movie S1) suggests the presence of three clusters of DoS that, while connected by other residues that (to a lesser extent) are also likely to contribute to specificity, are located on different patches of the kinase domain: cluster 1, while mainly containing residues from the bigger C-lobe (the lobe best described in terms of its importance for kinase specificity), also spans residues from the N-lobe and contacts directly with, and to a large degree encapsulates, the substrate peptide. This could be considered the main cluster directly driving specificity and includes several of the residues and structural features previously linked to specificity (e.g., the activation segment or the P+1 loop; Nolen et al., 2004), as well as new ones, such as the residues in the αC helix that we experimentally validated to encode specificity. Cluster 2, on the other hand, is comparably smaller and contains exclusively residues belonging to the big C-lobe of the domain. Given its position, we suggest that this cluster of residues could affect specificity by closing (or opening) the domain inward (or outward), effectively modifying the size and shape of the binding pocket, especially on the region that contacts the N-terminal section of the substrate peptide. Finally, cluster 3, containing very few residues of the small N-lobe, seems to contribute to specificity by causing subtle structural re-arrangements leading to differences in the opening and closing of the lobe onto the peptide. Overall, while all three clusters simultaneously encode specificity on different parts of the substrate peptide, by shaping the active site in a cumulative and non-linear fashion, cluster 1 appears to be the main driver of specificity (Figure S4).

### Domain and Specificity Evolution

**Figure 5:**
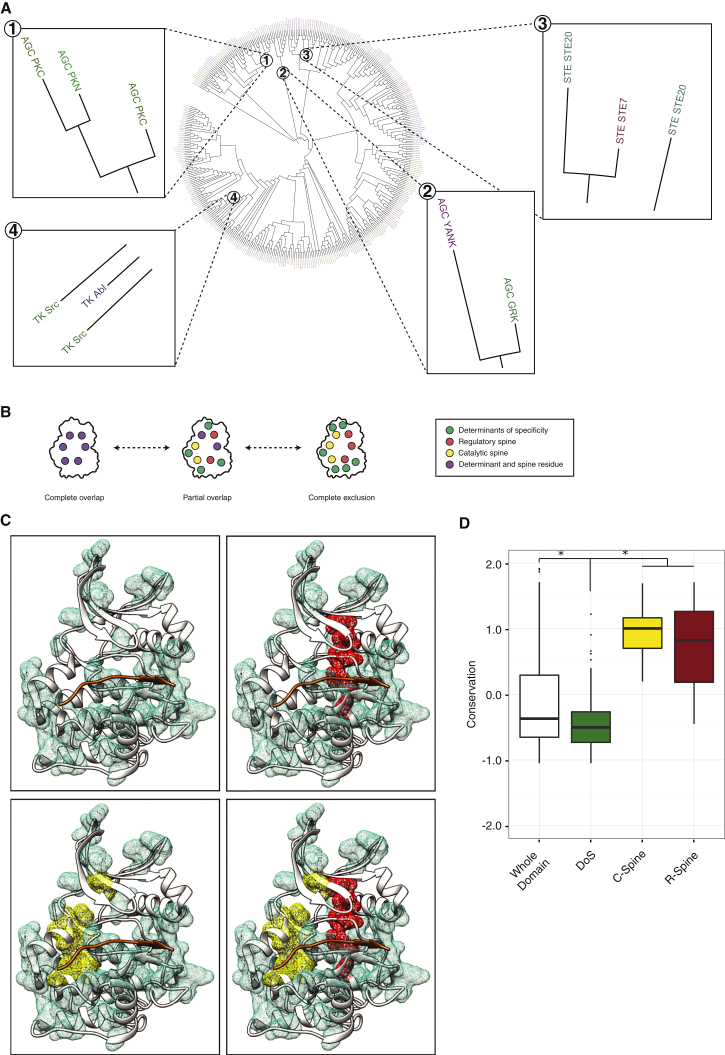
Evolutionary Aspects of DoS and Their Co-existence with Kinase Spines (A) As can be observed from the different panels on this DoS-based Dendrogram, where several kinases are localized discordantly with whole-domain evolution, peptide specificity evolution cannot be directly inferred from whole-domain specificity. These differences highlight how kinases have accumulated mutations on these specific residues, i.e., DoS, in order to evolve different specificities. For further explanation and information, please refer to Experimental Procedures and Figures S5. (B) We next investigated how DoS co-evolved with residues involved in structural changes related to catalysis (kinase spines). As can be seen here, there are different possible degrees to which DoS and spines could co-exist, ranging from complete overlap (left) to complete exclusion (right). In (C), we investigate which of these models is more supported by our data. (C) By comparing the relative localization of the DoS (top-left structure) together with the residues belonging to the catalytic spine (in yellow, bottom-left structure), the regulatory spine (in red, top-right structure) or all residues together (bottom-right structure), our data suggest that the subgroups of residues that are DoS or spines are mutually exclusive or, in other words, that residues classified as DoS are not part of the catalytic or regulatory spines. Like in Figure 4A, the structure used is that of Akt/PKB in complex with GSK3 peptide (PDB ID: 1O6K; Yang et al., 2002). (D) Evolutionary conservation for the different subsets of residues (whole domain, DoS, C-spine, and R-spine) was computed as the negative of entropy, using AL2CO algorithm with its default parameters (50), and shown to be significantly lower in DoS compared to the whole domain and the spines (p = 0.014 and p = 1.4 × 10^−6^ using Wilcoxon test, respectively).

**Figure S5:**
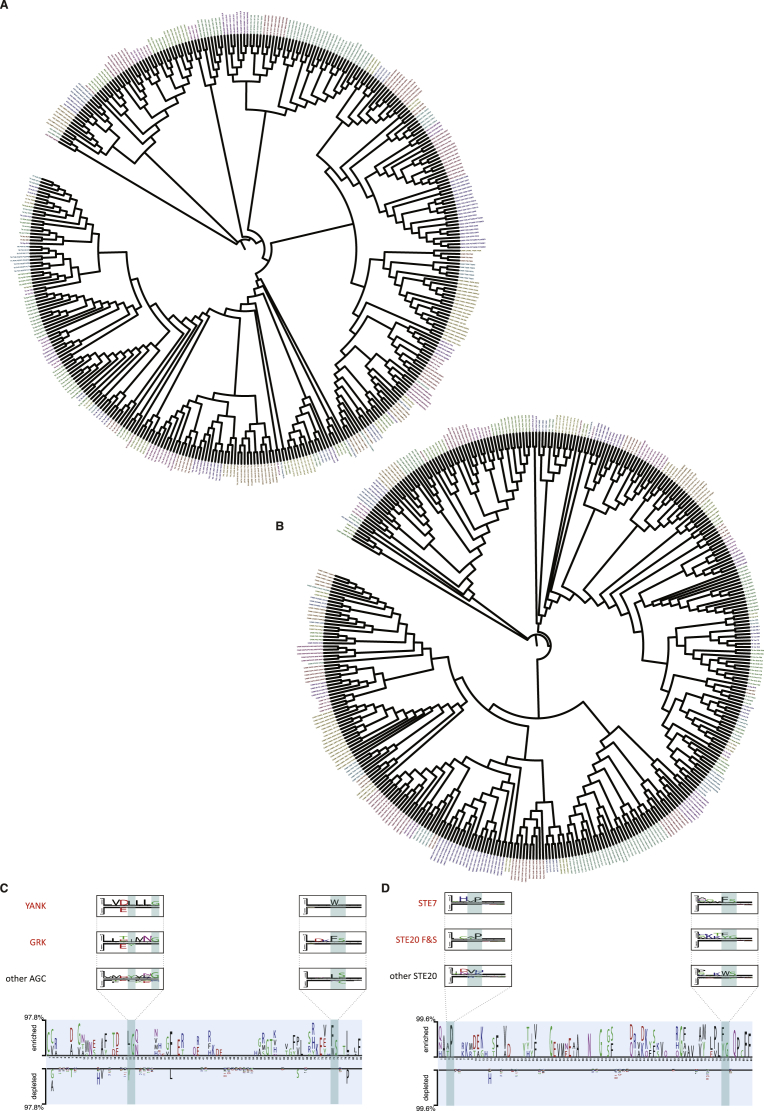
Related to Figure 5 (A) DoS-Based Evolutionary Tree. By producing a sequence alignment including only positions identified as determinants of specificity by KINspect, we could explore how kinase domain specificity evolved (as compared to the evolution of the domain as a whole, B). Several examples of significant differences between the domain-wide and DoS-centric evolutionary trees (as shown in Figure 5) confirm how these two evolutionary paths, of domain overall function and specificity, are not completely coupled. This figure was created with iTOL (http://itol.embl.de; Letunic and Bork, 2007). (B) Whole-Domain Evolutionary Tree. For comparison purposes, as explained in Figures 5, we built a domain-wide evolutionary tree following the same strategy as in the case of the DoS-based tree, but taking into consideration all residues within the kinase domain. (C and D) DoS and Motif Logos that Cause Kinase Family Re-arrangements in the DoS-based tree. Further exploration of the DoS and DoS-centered alignment clarifies the reasons why there are marked differences between the DoS-based tree and the one where the whole domain was considered. In the case of Yank and Grk families, for instance, a preference for Leucine (L) over Tyrosine (Y) in the first highlighted DoS and for large hydrophobic amino-acids (as opposed to Leucine) in the other one, exemplify why they cluster separately from other AGC kinases. In the case of the STE7 family embedding within the STE20 family, a couple of strong preferences for Proline (P) and Phenylalanine (F) in the two highlighted DoS provide evidence for the clustering of kinases belonging to two different families (i.e., STE7 and STE20’s STLKs and FRAYs). For further details on how these logos were built please refer to Supplemental Experimental Procedures.

We next set out to explore whether evolutionary insights could be derived from these results. It has previously been observed that the evolution of the kinase domain as a whole is not an accurate reflection of how different kinases have evolved different peptide specificities (Miller et al., 2008, Rausell et al., 2010). Thus, we speculated that a Dendrogram based solely on residues identified as DoS by KINspect could carry significant differences compared to a domain-wide phylogenetic tree. Indeed, Figure 5A (and Figure S5) illustrates how the relationships between kinases (and even between kinase families) appear to deviate when addressed from the DoS’ perspective. This DoS-based tree (Figures 5A and S5) illustrates interesting differences including: (1) the embedding of kinase families within other families, such as in the case of the PKN family, embedded within the PKC family, (2) clustering of seemingly unrelated families, such as the Yank and GRK families, or (3) the splitting of families in two sets displaying marked amino acid differences on their DoS, such as in the case of the Ste20 family.

Thus, this analysis provides further proof and explanation as to how and why the evolution of the entire domain does not always parallel specificity evolution (Capra et al., 2012). Using the DoS-based Dendrogram (based on the DoS residues predicted by KINspect), we have provided an alternative evolutionary explanation of the human kinome, which we argue, more accurately reflects functional diversity and specificity evolution. Such a view, of proteins evolving new specificities by diverging at specific sites within protein domains, is supported by other recent studies conducted on bacterial signaling networks (Capra et al., 2012, Skerker et al., 2008).

### Kinase Specificity, Regulation, and Activity Are Loaded onto Different Residues

With the aim of interpreting our results from a more global perspective, we investigated to what extent the DoS residues identified by KINspect can interplay with residues known to be involved in the catalytic activation and regulation of the kinase domain.

Two independent sets of residues playing such crucial roles have been identified forming hydrophobic interactions at the core of the domain and stabilizing the active conformation of the domain (Kornev et al., 2006, Kornev et al., 2008). These two networks of residues, critical for activation and regulation, are named the catalytic and regulatory spines, respectively. In order to examine how the DoS interact with the two spines (Figure 5B), we visualized the residues forming the catalytic and regulatory spines as well as those identified as DoS in the same kinase structure (Figure 5C). This representation shows that both groups are virtually mutually exclusive, with kinase domain residues belonging to either spines or the DoS set (mostly localized on the surface of the domain), but rarely both.

Despite this apparent separation of biological functions in the kinase domain, it is at the same time equally important to highlight that KINspect, in agreement with previous observations (Nolen et al., 2004), identifies the activation segment as playing a critical role in specificity. Since this segment also plays a crucial role in regulation and catalysis by stabilizing the R-spine (Kornev et al., 2006, Kornev et al., 2008), in spite of the apparent general decoupling of these different functions, on this particular segment, they still appear to be partially intertwined (Figure 5C). Moreover, highlighting the distinct evolutionary and functional paths of these sets of residues, we could quantify their differences in sequence conservation and conclude that DoS are typically residues with considerably lower conservation than the highly conserved spines and many other residues in the domain (Figure 5D).

### Similarly Sparse Networks of Determinants Drive Specificity in the SH2 Domain

**Figure 6:**
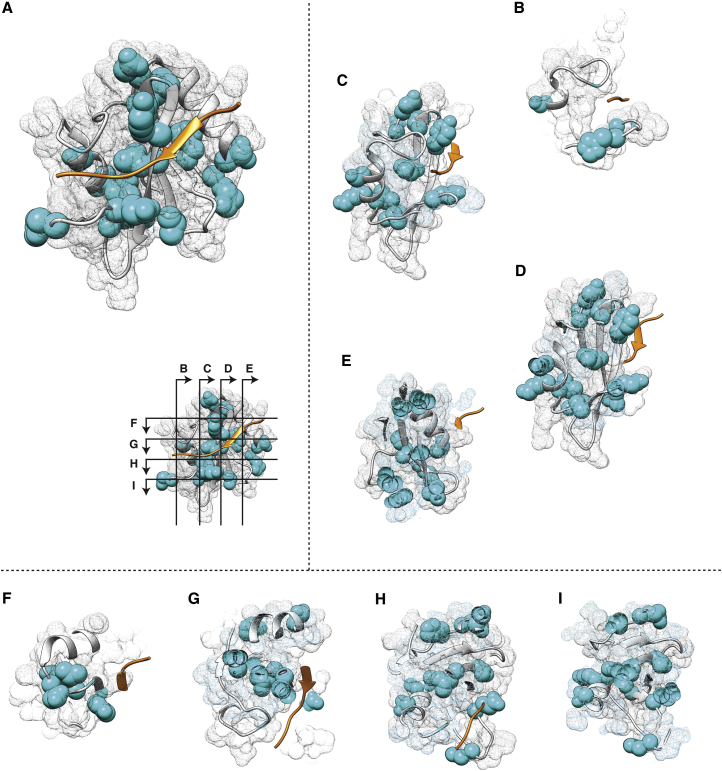
Determinants of Specificity in the Human SH2 Domain (A) Mesh representation of the SH2 domain, including its secondary structure in cartoon representation and a bound peptide substrate colored in orange. Positions predicted as DoS by KINspect (i.e., residues with a KINspect score higher than 0.9) are highlighted in cyan. (B–I) As in the case of the kinase domain, longitudinal (B–E) and transversal (F–I) slices were taken through the SH2 domain at the planes indicated in the inset in (A). For a dynamic visualization of this structure, please refer to Movie S2. The structure used is that of SAP in complex with SLAM peptide (PDB ID: 1D4T; Poy et al., 1999).

**Figure S6:**
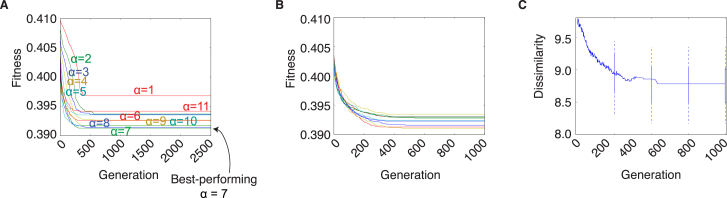
Related to Figure 6 (A) Alpha Determination for SH2 KINspect. Similarly as in Figure S2, an optimal parameter ‘alpha’ (α) needed to be set for KINspect when deployed on the SH2 dataset. As shown here, in this case, the best-performing value is α = 7, so this value was used subsequently. (B) Fitness Evolution for SH2 KINspect. As observed in Figure S2 for the kinase domain, fitness evolutions and convergences are similar for the ten different evaluation runs of SH2 KINspect. (C) SH2 Specificity Masks Similarity Over the Different Generations. Similarly as in Figure S2 for the kinase domain, an increase in similarity among specificity masks between different evaluation runs is observed as we move closer to the end of the KINspect optimization, suggesting different evaluation runs converge approximately to the same solution.

To investigate the generality of these observations, we explored DoS patterns in another signaling modular protein domain, namely, the SH2 domain. Following a very similar approach as described for the kinase domain, and after identifying the required parameters (Figure S6) appropriately, KINspect identified several SH2 residues that are likely involved in peptide specificity (Figure 6; Movie S2).

Being a smaller domain of typically approximately 100 residues (as can be appreciated in the SH2 domain alignment in Data S4) and generally showing less variability in peptide specificity, it is perhaps not surprising that KINspect converged considerably faster for the SH2 domain (Figure S6) than in the case of the kinase domain.

Despite this difference, as with the kinase domain, independent deployments of KINspect led to the highly reproducible results (Figure S6), and the general model of peptide specificity observed in the kinase domain, where a sparse network of DoS involving a relatively larger number of residues, was also observed in the case of the SH2 domain (Figure 6; Data S5). Similarly, whereas some DoS were close to the peptide (e.g., Figures 6C, 6D, and 6G), others were relatively far away from it (e.g., Figures 6E and 6I), though often connected by inter-residue “canals.” The aforementioned control experiments, where uniform and randomized domain-specificity sets were used (Figure S3), exclude the possibility that the similarities between these results for the kinase and SH2 domains emanate from some intrinsic bias in our computational approach. The spatial representation for several of our DoS is also supported by previous studies of SH2 domains (Halabi et al., 2009, Lenaerts et al., 2008). All in all, this suggests that our findings, with a high number of DoS residues located away from the substrate, far from being unique to kinase specificity could be a more general trend applicable to other modular protein domains (Tompa et al., 2014).

## Discussion

**Figure 7:**
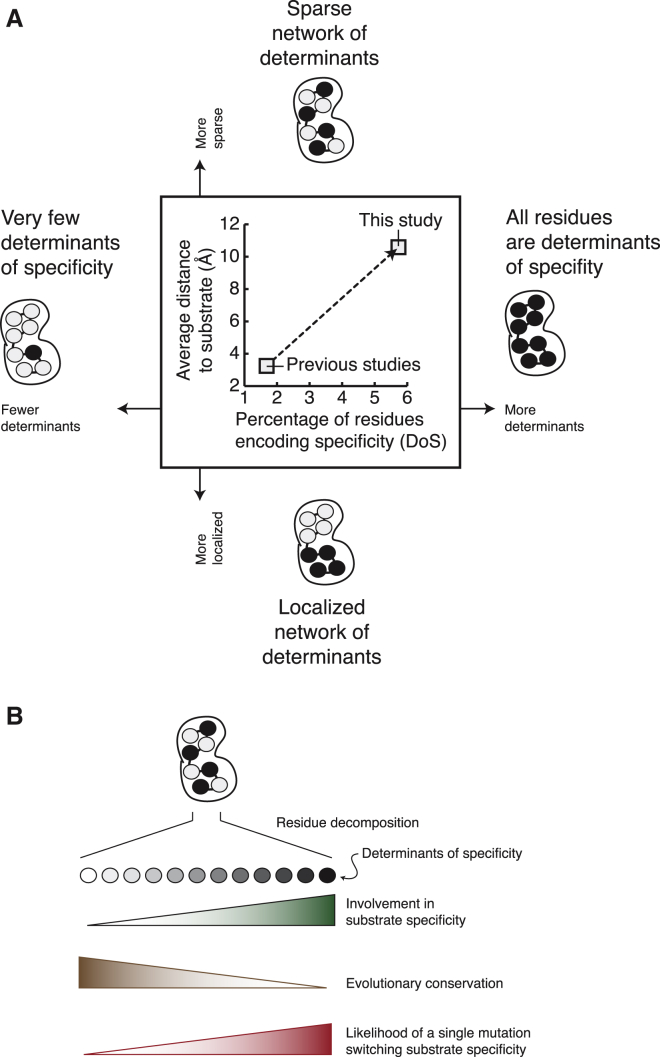
Model for DoS Encoding Specificity and Mutations on DoS Perturbing Substrate Specificity (A) By computing the percentage of residues encoding specificity (DoS) and their average distance to the substrate, we conclude that the set of DoS uncovered by KINspect represent a larger and more sparse group of residues and that residues can contribute to peptide specificity even if they are not located in close proximity to the substrate. (B) In this article, we have described the degree to which each residue contribute to specificity on a more quantitative scale and experimentally validated three novel determinants of specificity (Figure 3). Our results also indicate that specificity is loaded onto a set of residues that is less conserved than most others in the domain and where mutations, in cancer or evolution, can easily cause substrate specificity switches by single mutations. As shown in similar signaling systems (Skerker et al., 2008), such mutations represent key turns in how different kinases evolve and occupy separate and sometimes overlapping substrate subspaces. Similar mutations have been seen in cancer (Borrello et al., 1995, Creixell et al., 2012, Santoro et al., 1995, Songyang et al., 1995), despite the fact that this type of mutations has been largely understudied, and their role in the disease remains largely unknown (see Creixell et al., 2015).

Despite the crucial importance of signaling fidelity in biological organization and cellular responses to environmental cues, our perception of how peptide specificity is encoded in the kinase domain has been highly fragmented and biased toward certain kinase families, non-human species, or a subset of kinase domain residues (e.g., those close to the peptide substrate). Here, we developed a data-driven systematic approach to investigate the presence of DoS residues throughout the human kinome, experimentally validated several of these DoS, which together with those shown in the accompanying article (Creixell et al., 2015) encode specificity for the five residue positions most critical for specificity in the peptide substrate (P-3, P-2, P0, P+1, P+2), and identified a distributed, but interconnected, network of DoS in different parts of the kinase domain. In contrast to previous studies, our results suggest specificity is driven by a larger number of residues and a more distributed network of typically non-conserved sets of residues than previously appreciated (Figures 7A and 7B).

### Determinants in the Context of Spines and Sectors

The sparse networks of DoS also present interesting implications when compared and contrasted with previous work.

First, as mentioned earlier and illustrated in Figure 5, we note an apparent discrepancy between the residues we identify as DoS, mostly localized on the surface of the domain, and the core residues that form the catalytic and regulatory spines (Kornev et al., 2006, Kornev et al., 2008). Whereas this suggests some degree of functional and evolutionary separation between catalytic activity (and regulation thereof) and peptide specificity, a separation of functions that is similar to those employed in other signaling systems (Goldman et al., 2014), our results also indicate that the activation segment provides a link between these biological functions. The fact that different functions seem to be “co-loaded” on this segment could explain why a large fraction of cancer mutations perturb this critical part of the kinase domain (Dixit et al., 2009, Creixell et al., 2015).

Moreover, this separation of function, together with our finding of very different evolutionary speeds and trajectories for spines and DoS, makes us speculate that kinases have evolved within tight constraints around spines, where maintaining spine integrity was critical to retain kinase activity. On the other hand, the more loose constraints on DoS have facilitated the evolution of new kinases with distinct specificities, a view that is consistent with the current understanding of the evolution of signaling systems (Lim and Pawson, 2010).

Furthermore, the picture portrayed by our results of sparse networks of multiple residues driving specificity together would fit within the scope of more recent theories on protein function, namely, the so-called protein sector model. According to this model, protein function is often encoded in protein sectors, defined as subsets of co-evolving residues (Halabi et al., 2009, Lockless and Ranganathan, 1999) identified in different protein domains, which often also include long-range interactions between distant residues by allosteric regulation (Reynolds et al., 2011). Our results suggest that similar mechanisms could be at work determining specificity in both the kinase and SH2 domains.

### Perspectives

Despite the significant conceptual and analytical leap forward provided by KINspect in terms of capability and coverage, continued experimental and computational advances will make it even more precise and accurate in the future.

From an experimental perspective, it is clear that obtaining peptide specificity profiles for a larger number of kinases (currently, the percentage of kinases for which their specificity has been profiled is only about 30% of the whole human kinome) will only improve our method’s results.

In terms of extending to other applications and expanding our current approach, KINspect's methodology could potentially be applied to several other fundamental biological questions such as the identification of residues driving kinase inhibitor binding and specificity. Naturally, we also plan to expand KINspect to add new peptide-recognizing modular domains other than the already-included kinase and SH2 domains (e.g., SH3 or WW domains) or even include inter-positional dependencies within the substrate peptide in the future when data become available.

### Implications for Evolution and Disease

As introduced earlier, peptide specificity is a crucial component of a wider cellular requirement, signal fidelity, which ensures that cells will correctly decode input cues and respond accordingly. Changes in this system have been identified as playing a critical role in multicellular metazoan evolution (Tan et al., 2009, Tan et al., 2011), but also, at the domain level, in how proteins evolve new specificities allowing cells to start responding to new cues or unfold new responses to them (Capra et al., 2012, Marengere et al., 1994, Skerker et al., 2008, Zarrinpar et al., 2003). While this has perhaps been less studied in a disease context, it has been suggested that the same process occurs in cancer (Borrello et al., 1995, Santoro et al., 1995, Songyang et al., 1995). In the accompanying article (Creixell et al., 2015), we utilize the bona fide DoS described here to identify cancer mutations perturbing them and experimentally validate their role in causing signaling rewiring (Creixell et al., 2012) and thus contributing to oncogenesis by affecting kinase specificity. We are optimistic these mutations, and new ones that will be identified in the future, will constitute a novel and solid foundation for enhanced appreciation of how signaling networks are perturbed in cancer and other diseases.

## Experimental Procedures

### Learning Classifier System

The learning classifier system briefly described in the main text that constitutes the computational engine behind KINspect is illustrated in Figure 2. Further algorithmic and mathematical details can be found in Supplemental Experimental Procedures.

### Frobenius Distance between Matrices or Vectors

As a measure of dissimilarity between matrices or vectors, the Frobenius distance or norm can be simply calculated as the square root of the difference between every value in the two matrices or vectors squared (Ellis and Kobe, 2011).

### Domain Information and Alignments

Domain sequences for all human kinase domains and additional information on the human kinome were obtained from the http://kinase.com/ repository, with more recent and up-to-date unpublished data kindly provided by Dr. Gerard Manning (G. Manning, personal communication; Manning et al., 2002). Similar sequence and domain information was obtained for all the human SH2 domains from the SH2 domain site (Liu et al., 2006). Sequences were aligned using ClustalW2 (Larkin et al., 2007), and alignments were further refined manually with help from Dr. Toby Gibson (EMBL).

### Dendrogram Construction

Distance matrices between kinases were computed using BLOSUM62 substitution matrix (Henikoff and Henikoff, 1992). The distances in the kinome tree are based on all the columns in the alignment, while the distances in the specificity tree only consider the selected DoS columns in the alignment. We used neighbor joining to build both trees.

### Computing Minimum Distance to Substrate from PDB Files

In a similar manner as described in the accompanying article (Creixell et al., 2015), we computed a measure of the minimum distance between any position in our alignment and the substrate peptide. This distance was obtained by extracting distance information from ten representative kinase-substrate structures deposited in PDB (AKT2 [PDB ID: 1O6K]; Yang et al., 2002, PIM1 [PDB ID: 2BZK]; Bullock et al., 2005, DYRK1A [PDB ID: 2WO6]; Soundararajan et al., 2013, CDK2 [PDB ID: 2CCI]; Cheng et al., 2006, PAK4 [PDB ID: 2Q0N]; Chen et al., 2014, EPHA3 [PDB ID: 3FXX]; Davis et al., 2009, FES [PDB ID: 3CD3]; Filippakopoulos et al., 2008, EGFR [PDB ID: 2GS6]; Zhang et al., 2006, IGF1R [PDB ID: 1K3A]; Favelyukis et al., 2001, INSR [PDB ID: 3BU3]; Wu et al., 2008). By developing and deploying in-house python scripts that utilize the biopython package Bio.PDB, we could extract distance features between every residue of these kinase-substrate pairs. Subsequently, this information was collected and, by using the alignment to track the same position on different kinase-substrate structures, the minimum distance for each alignment position was obtained. Additional information on substrate peptide distance for the different mask positions can be found in Data S3.

### PSPL Analysis

PKCγ (WT and mutant) was produced in HEK293T cells with a 3 × FLAG epitope tag at the C terminus and isolated by affinity purification on M2 FLAG antibody resin (Sigma-Aldrich) as described (Mok et al., 2010). Pim1 (WT and mutant) was expressed as an N-terminally hexahistidine-tagged fusion protein in *E. coli* and purified from lysates using TALON resin (Clontech). Peptide library analysis was performed by arraying a set of 182 peptide mixtures (50 μM) in a 1,536-well plate in kinase reaction buffer (2 μl/well). Buffer for Pim1 reactions was 50 mM HEPES (pH 7.4), 10 mM MgCl_2_, 0.1% Tween 20, and buffer for PKCγ reactions was 50 mM Tris-HCl (pH 7.5), 10 mM MgCl2, 1 mM DTT, 0.1% Tween 20 containing a 5-fold dilution of lipid activator (EMD Millipore). Peptides had the sequence Y-A-X-X-X-X-X-S/T-X-X-X-X-A-G-K-K-biotin, in which X positions were generally an equimolar mixture of the 17 amino acids excluding Ser, Thr, and Cys, and S/T is an even mixture of Ser and Thr. In each well of the array, the peptide had one of the 20 amino acids fixed at one of the nine X positions. In addition, two peptides were included that fixed either Ser or Thr at the phosphoacceptor position. Reactions were initiated by adding kinase (to 8 μg/ml) and [γ−^33^P]ATP (50 μM at 0.03 μCi/μl), incubated 2 hr at 30°C, and then 200-nl aliquots were transferred to a streptavidin membrane (Promega). Membranes were washed and dried as described and exposed to a phosphor screen. Radiolabel incorporation into each peptide mixture was quantified by phosphor imaging using QuantityOne software (Bio-Rad). Following background subtraction, data were normalized so that the average value for a given position within the peptide was equal to 1. Normalized data from two (PKCγ) or three (Pim1) separate runs were averaged, log2 transformed, and converted to heatmaps in Microsoft Excel.

## Author Contributions

P.C. and R.L. conceived the project. P.C., A.P., C.C.S., and M.N. developed and implemented the computational framework. P.C., C.J.M., H.J.L., and B.E.T. devised and/or performed experiments. P.C. and A.P. generated the structural visualizations with Chimera and performed the evolutionary analysis of DoS. R.L. oversaw the project. P.C. and R.L. wrote the article assisted by the other authors. All the authors read and approved the final manuscript.

## Supplemental Information

Document S1. Supplemental Experimental Procedures

Table S1. Table of Previously Reported Determinants of Specificity, Related to Figure 3

Data S1. Kinase Domain Sequence Alignment, Related to Figure 2 and Experimental Procedures

Data S2. Kinase KINspect Results (Average over Ten KINspect Evaluation Runs), Related to Figures 2, 3, and 4

Data S3. Additional Information on the Kinase KINspect Results Regarding Substrate Peptide Proximity, Related to Figure 4

Data S4. SH2 Domain Sequence Alignment, Related to Figure 6 and Experimental Procedures

Data S5. SH2 KINspect Results (Average over Ten KINspect Evaluation Runs), Related to Figure 6

Movie S1. Structural Representation of the Determinants of Specificity in the Kinase Domain, Related to Figure 4

Movie S2. Structural Representation of the Determinants of Specificity in the SH2 Domain, Related to Figure 6

Document S2. Article plus Supplemental Information
