# Supplementary material for: Unmasking Determinants of Specificity in the Human Kinome
Source: Cell. 2015 Sep 24;163(1):187–201. doi: 10.1016/j.cell.2015.08.057 (PMC4644237; doi:10.1016/j.cell.2015.08.057)
Supplement: Document S1. Supplemental Experimental Procedures [file mmc1.pdf]

Cell

Supplemental Information

Unmasking Determinants of Specificity in the Human Kinome

Pau Creixell, Antonio Palmeri, Chad J. Miller, Hua Jane Lou, Cristina C. Santini, Morten Nielsen, Benjamin E. Turk, and Rune Linding

SUPPLEMENTAL EXPERIMENTAL PROCEDURES

Detailed Description of the KINspect Methodology Illustrated in Figure 2

In step 1, i.e. the prediction step, every specificity mask is used to compute the peptide specificity of each kinase (defined by PSSMs, Figure S1). There are two main aspects that intervene in the final PSSM predictions; a) for each kinase for which we want to predict its specificity (i.e. the query kinase), any other kinase belonging to the same kinase family will be excluded from the training set (Manning et al., 2002), in a process that ensures our method stays global and does not over-fit; and b) after comparing, using our kinase domain alignment (File S1), the query kinase to all other kinases in the training set, the specificity mask is used to boost the similarity score of those kinases that resemble the query in positions that are deemed important for specificity by the mask and drop the similarity score of other kinases that are similar on positions deemed unimportant. The mask-weighted similarity score of each kinase in the training is then integrated with its experimentally determined/observed PSSM, so that the predicted PSSM for the query kinase will more closely resemble those kinases that were similar to it in positions deemed most relevant for specificity by the mask (for further details, please refer to Experimental Procedures). This integration critically depends on a parameter, alpha (**α**), which determines the "democratic" or "autocratic" integration of more similar or dissimilar domains, which has to be optimized to each domain-peptide case (Figure S2). Naturally, this first step is repeated over all kinases used as query and, subsequently, over all 100 specificity masks that form a set.

In step 2, i.e. fitness evaluation (Figure 2), the system simply evaluates the predictive performance of each mask by comparing the computationally predicted PSSMs with the experimentally-derived PSSMs, and fitness is calculated as the Frobenius distance between the two matrices (Experimental Procedures).

In step 3 (Figure 2), the generation of a new set of specificity masks is produced by: a) selecting the best-performing masks from the last generation, the so-called 'elite' subpopulation, and generating new variations of these elite masks by b) mutation (where a few specific positions are converted onto other random values) and c) cross-over (where segments of two distinct elite masks are combined onto a new mask). After scanning a range of values, the elite, mutation and cross-over fraction parameters were set to 0.20, 0.70 and 0.80, respectively. The algorithm is defined in formal computational and mathematical terms below.

Algorithmic and Mathematical Description of the Learning Classifier System within KINspect

During the prediction step of the KINspect method, three equations define the behavior of the method. The first describes how similarity between kinases is calculated:

$$Sim(KIN1, KIN2) = \sum_{x=1}^{N} \frac{S(KIN1x, KIN2x)}{\sqrt{S(KIN1x, KIN1x) \cdot S(KIN2x, KIN2x)}} SSx$$

where ***x*** is one position in the kinase domain alignment, ***S(KIN1x,KIN2x)*** would be the similarity score between the residues of ***KIN1*** and ***KIN2*** in this position ***x***, as determined by a substitution matrix of choice (e.g. BLOSUM62 (6)) and ***SSx*** is the specificity score of position ***x*** as determined by the specificity mask. Finally, ***N*** is the total number of positions in the domain alignment. As introduced in the main text, by incorporating the specificity score, we achieve a reinforcement of residues deemed important for specificity and dilution of residues deemed less important for specificity. This part of the method is similar to, and represents a generalization of, the structure-based Pickpocket method (10).

Subsequently, a final mask-weighted similarity is produced using the following equation:

$$W_{KIN1} = \frac{(Sim(KIN1, KIN2))^{\alpha}}{\sum_{KIN1=A}^{L} (Sim(KIN1, KIN2))^{\alpha}}$$

where ***L*** represents all the training kinases that will be used to assess ***KIN2*** and α represents the parameter that establishes the importance of scoring high similarity for the contribution towards specificity profiles' prediction, with low values of ***α*** meaning a more "democratic" contribution of every kinase, regardless of its similarity, and higher values of ***α*** leading to predictions driven by the most similar kinase. Figure S4 illustrates our exploration of this parameter and identification of its best value for our specific problem.

Once the final mask-weighted similarity has been determined, the predicted PSSM or specificity profile is generated simply as described in this equation:

$$PSSMpred_{KIN2} = \sum_{KIN1=A}^{L} W_{KIN1} \cdot PSSMobs_{KIN1}$$

where ***PSSMpredKIN2*** would be the new predicted PSSM and ***PSSMobsKIN1*** represents the observed (i.e. experimentally determined) PSSM.

We note that KINspect can also be considered as a kernel-based regression method where the specificity mask and the alpha-parameter represent a set of hyper-parameters to be learned from data. We are currently investigating whether future versions can be implemented fully bayesian framework (Bishop, 2007). Similarly, we are working on experimental and computational procedures to overcome current limitations of PSSM profiles, such as its inability to capture coupling between residues at the substrate level. For an abstract representation of the KINspect method in pseudo-code form, please see below.

Logos

Each global specificity-cluster logo shown in Figure S5 is built from all the DoS columns in the kinase alignment, selecting as a positive set the families or subfamilies of interest, and as background the remaining kinases belonging to kinase groups which are different from those found in the positive set. The local specificity-cluster logos are built from sequence windows in the domain-wide alignment of +/-3 residues around DoS columns, using the same strategy for positive and negative sets as before. All logos were built with Two Sample Logo (Vacic et al., 2006).

Pseudo-code for KINspect (implemented in Python)

KINspect Algorithm

Input: **K =** Training Set Kinases
MSA = Multiple Sequence Alignment of Training Set Kinases, with C residue positions
PSSMobs = Dictionary of PSSM matrices, corresponding to the Training Set kinases

00: Start with a pool of random masks, $m \in M$, such that every m has C positions, and $m_c \in [0,1]$
01: For each mask, $m \in M$:
02: For each query kinase, $k \in K$:
03: Define a subset of kinases, $S_k \subseteq K$, excluding kinases belonging to the same family as k
04: Call subroutine PSSM Prediction with input: k, Sk, m, MSA, PSSMobs, to obtain PSSMpred (k)
05: Let Dk = Frobenius Distance between PSSMpred (k) and PSSMobs (k) for kinase k
06: Let Fm = Fitness for mask m, summing D_k over all query kinases $k \in K$
07: Order the masks according to F_m, and select, as elite, the 20% top ranked masks
08: Generate a new pool of masks M, performing crossing overs and mutations within the elite masks population
09: Repeat 01-08 until convergence
10: Return the top ranked mask found in step 07.

Subroutine PSSM Prediction

Input: **k =** query kinase
S_k = list of kinases, subset of Training Set Kinases
m = mask
MSA = Multiple Sequence Alignment of Training Set Kinases
PSSMobs = dictionary of PSSM matrices, for the Training Set kinases
α = parameter increasing the importance of PSSM profile contributions from training kinases having higher similarity with query

00: For each kinase $s \in S_k$:
01: Call subroutine Sequence Similarity, with input: k, s, MSA, to compute $SeqSim_{k,s}$
02: Let Contribs = dot product between $SeqSim_{k,s}$ and m, elevated to α
03: Let W_{sum} = sum of Contribs over all $s \in S_k$
04: For each kinase s in Sk:
05: Let W_s = Contribs / W_{sum}
06: Let PSSMcontrib (s) = Ws * PSSMobs (s)
07: Let PSSMpred = sum PSSMcontrib (s), over all $s \in Sk$
08: Return PSSMpred

Subroutine Sequence Similarity

Input: **k =** query kinase
s = kinase for sequence comparison
MSA = Multiple Sequence Alignment of Training Set Kinases, containing sequences of k and s, with C columns
SM = Substitution Matrix

00: For each column $c \in C$ in MSA:
01: Let B_c = SM(MSA(k,c), MSA(s, c)) , i.e. the substitution value in column c, between the residue of kinase k and the residue of kinase s
02: Let P_c = SM(MSA(k,c), MSA(k, c)) * SM(MSA(s,c), MSA(s, c)) , i.e. the product between self-substitution values of k residue and s residue in column c
03: Compute $SeqSim_c$, dividing B_c, by the square root of P_c
04: Return SeqSim

SUPPLEMENTAL REFERENCES

Bishop, C. (2007). Pattern Recognition and Machine Learning (Information Science and Statistics).

Brinkworth, R.I., Breinl, R.A., and Kobe, B. (2003). Structural basis and prediction of substrate specificity in protein serine/threonine kinases. Proc Natl Acad Sci U S A *100*, 74–79.

Ellis, J.J., and Kobe, B. (2011). Predicting protein kinase specificity: Predikin update and performance in the DREAM4 challenge. PLoS One *6*, e21169.

Henikoff, S., and Henikoff, J.G. (1992). Amino acid substitution matrices from protein blocks. Proc Natl Acad Sci U S A *89*, 10915–10919.

Hutti, J.E., Jarrell, E.T., Chang, J.D., Abbott, D.W., Storz, P., Toker, A., Cantley, L.C., and Turk, B.E. (2004). A rapid method for determining protein kinase phosphorylation specificity. Nat Meth *1*, 27–29.

Letunic, I., and Bork, P. (2007). Interactive Tree Of Life (iTOL): an online tool for phylogenetic tree display and annotation. Bioinformatics *23*, 127–128.

Manning, G., Whyte, D.B., Martinez, R., Hunter, T., and Sudarsanam, S. (2002). The Protein Kinase Complement of the Human Genome. Science *298*, 1912–1934.

Miller, M.L., Jensen, L.J., Diella, F., Jørgensen, C., Tinti, M., Li, L., Hsiung, M., Parker, S.A., Bordeaux, J., Sicheritz-Ponten, T., et al. (2008). Linear motif atlas for phosphorylation-dependent signaling. Sci Signal *1*, ra2+.

Vacic, V., Iakoucheva, L.M., and Radivojac, P. (2006). Two Sample Logo: a graphical representation of the differences between two sets of sequence alignments. Bioinformatics *22*, 1536–1537.
