# Supplementary figures and images for: Unmasking Determinants of Specificity in the Human Kinome
Source: Cell. 2015 Sep 24;163(1):187–201. doi: 10.1016/j.cell.2015.08.057 (PMC4644237; doi:10.1016/j.cell.2015.08.057)

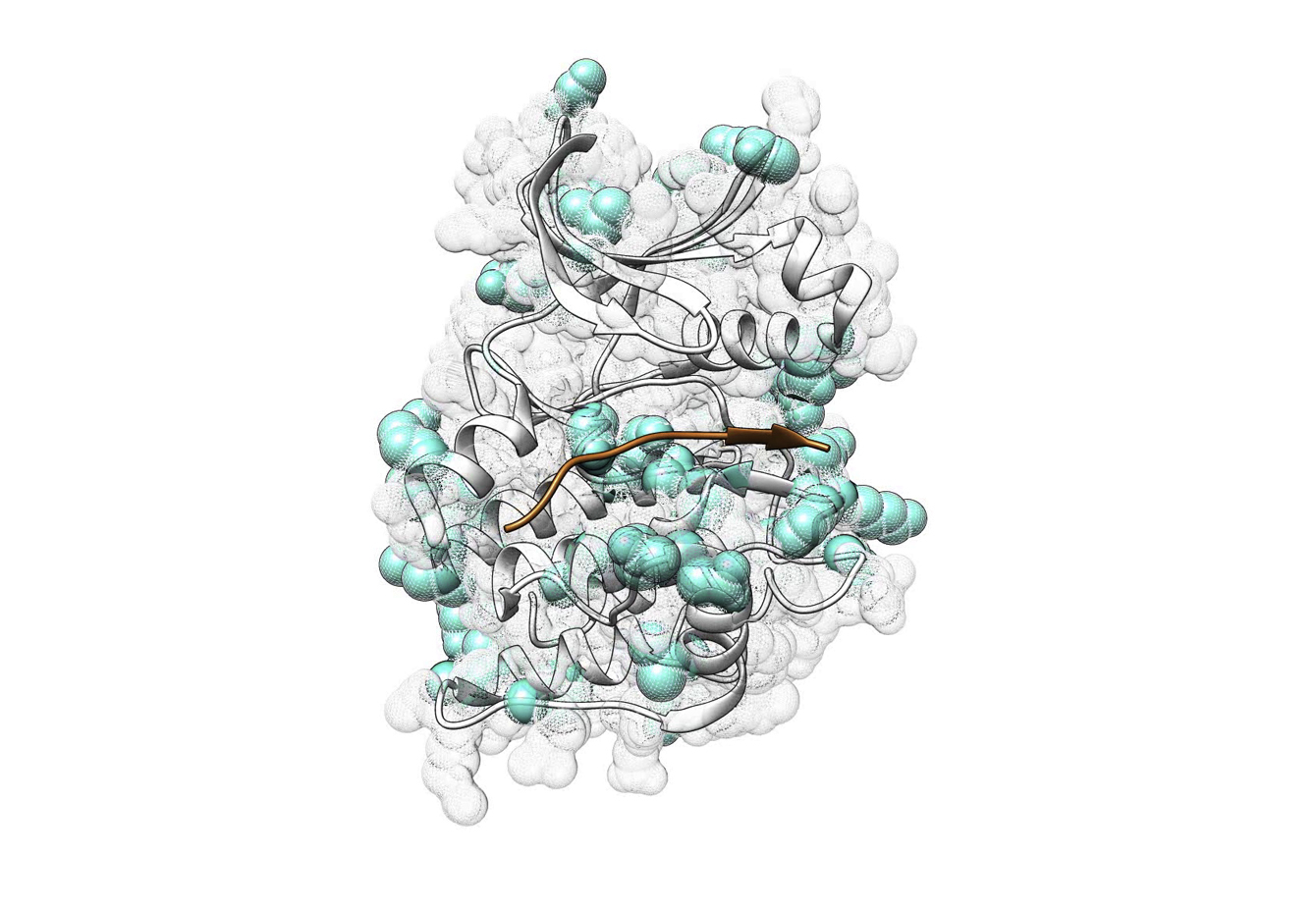

Supplement: Movie S1. Structural Representation of the Determinants of Specificity in the Kinase Domain, Related to Figure 4 [file mmc8.jpg]

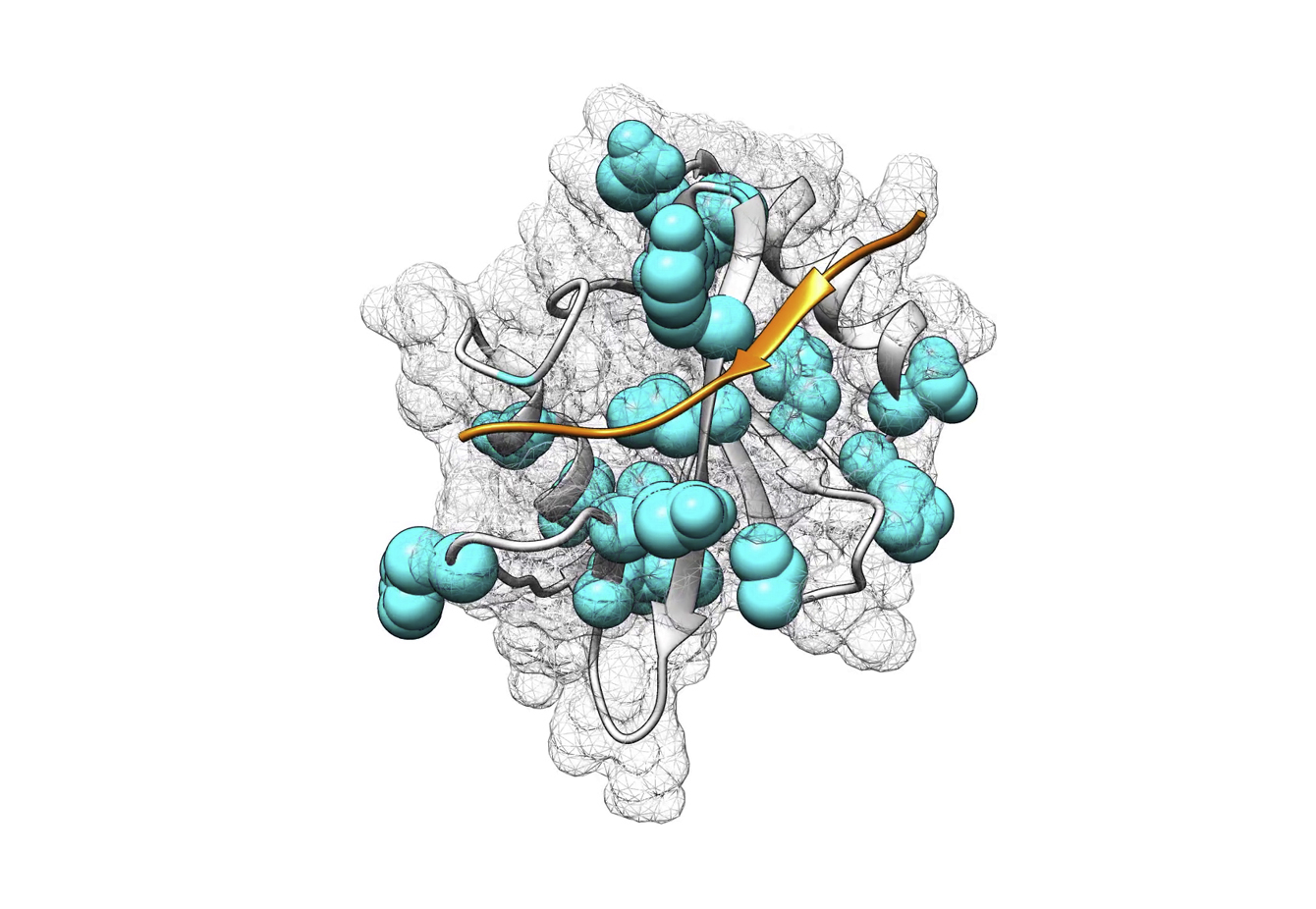

Supplement: Movie S2. Structural Representation of the Determinants of Specificity in the SH2 Domain, Related to Figure 6 [file mmc9.jpg]
